# Clinical practice guidelines in cancer: the European perspective

**DOI:** 10.1054/bjoc.2001.1773

**Published:** 2001-05

**Authors:** G Storme

**Affiliations:** OC AZ-VUB, Laarbeeklaan 101, Brussels, 1090, Belgium


*British Journal of Cancer* (2001) **84** (Supplement 2), 6–7
© 2001 FNCLCC
doi: 10.1054/ bjoc.2001.1773, available online at http://www.idealibrary.com on IDEAL®

### Clinical practice guidelines in cancer: the European perspective

**G Storme**

OC AZ-VUB, Laarbeeklaan, 101, 1090-Brussels, Belgium

The Organization of European Cancer Institutes (OECI) is a non-governmental, non-profit organization founded in Vienna in 1979. Initially, its main objectives were to improve communication between European cancer institutions and to increase collaboration between them. Evolving healthcare programs over the past 10 years have given the OECI the opportunity to take up new challenges. The expertise of members within the organization has enabled the OECI to address the problem of quality in cancer care on a European scale and to develop initiatives to improve the overall management and outcome of cancer patients. The assembly of the OECI has subsequently been able to set up specific working groups on the following topics: clinical practice guidelines, data monitoring and evaluation, economic evaluation, pre-clinical and clinical research, medical education, communication with cancer patients and the use of information technology and telemedecine. Within the context of cancer care, many of these approaches have already been shown to be important in routine daily practice, not only within specialized cancer centres, but also across all centres and institutions involved in cancer care. The OECI provides an excellent forum to pursue research in these areas and bids for research funds from the European Community (Organization of European Cancer Institutes, 2000).

In 1996, Andre Kuwalowski, the former president of the OECI asked the members for topics they particularly wanted to address. The development and dissemination of clinical practice guidelines was identified as a topic of high priority and one that is essential for improving the quality of care of cancer patients. It is recognized that guidelines must be implemented within a larger project that includes quality control, to show that where they are empolyed, patient outcome is better (Langmark, 1997).

In order to avoid duplication of effort, the programme committee decided to adopt, disseminate and implement guidelines that had already been produced. It was initially planned to use the National Cancer Institute's comprehensive cancer database PDQ® as a starting point. However, it soon became clear that various European institutions had already developed their own guidelines at a local, regional or national level. The Standards, Options & Recommendations (SOR) project was developed by the French National Federation of Cancer Centers (FNCLCC) and the 20 French Comprehensive Cancer Centres (CRCC) in collaboration with specialists from French public universities, general hospitals, private clinics and scientific societies. The program deals specifically with guidelines in cancer care (Fédération Nationale des Centres de Lutte Contre le Cancer, 1998) that are developed according to explicit quality criteria. Critical appraisal of the available evidence by multidisciplinary expert groups is an approach highly relevant to clinical practice in oncology.

The SOR documents are published widely in print in the form of monographs and articles and in electronic form. A CD-ROM is available (Fédération Nationale des Centres de Lutte Contre le Cancer, 1998) covering 25 different cancers, with others under development.

As with other guideline development programs, the SOR project uses 'levels of evidence' to inform the user of the evidence on which the recommendations are based. The classification used by the SOR program distinguishes five levels of evidence and takes into account not only the type of study but also the accordance or disagreement between trials (Bailar, 1997 LeLorier et al, 1997). This emphasizes the need for explicit criteria to be able to critically appraise the evidence and for the transparent reporting of guidelines (Lee et al, 2000; Therasse et al, 2000). It also highlights the need for high quality research in the development of high quality guidelines and ensures there are no conflicting situations (Marshall, 2000). It is also clear that even in cancer research, guidelines are essential (United Kingdom Co-ordinating Committee on Cancer Research, 2000).

To date, the language of the French version has been a limiting factor. Sections of the SORs have been translated into English, however, and can thus be distributed throughout Europe. A transparent guideline development and reporting process is necessary for the use of SOR guidelines by the OECI and its member institutions. This is essential in order to assess to what extent the guidelines can be used across borders, and at which points in the development process specific local input is needed so that the guidelines are relevant for the setting in which they are to be used. A potential problem with respect to the use of these guidelines throughout Europe is that, where the evidence is weak, there can be sociocultural differences between countries. The next step must be to translate the guidelines into other European languages.

Cancer care based on clinical practice guidelines can contribute to an improvement in outcome for cancer patients (Organization of European Cancer Institutes, 2000) and may contribute to the reduction in the significant differences in outcome between different European countries (Quinn et al, 1998). Perhaps the most important feature of the SORs for future practice is that they provide clinical algorithms as an aid for clinicians managing different clinical situations in daily practice. Looking to the future however, the impact of guidelines on outcome can only be shown if all cancer cases are accurately registered (Dickman et al., 1999).

The OECI has decided to collaborate with the FNCLCC and CRCCs in the translation and publication of the SORs, to make them available to all OECI member institutions. The OECI will also contribute to help maintain a regular updating program. The OECI will distribute SOR guidelines to all members and collaborating institutions, which have as their ultimate target the improvement of cancer care.

### REFERENCES

Bailar JC (1997) The promise and problems of meta-analysis [editorial; comment] [see comments]. *N Engl J Med* **337**: 559–561

Dickman PW, Hakulinen T, Luostarinen T, Pukkala E, Sankila R, Soderman B and Teppo L (1999) Survival of cancer patients in Finland 1955–1994 [see comments]. *Acta Oncol* **38** (Suppl 12): 1–103

Fédération Nationale des Centres de Lutte Contre le Cancer (1998) Recommendations pour la pratique clinique en cancérologie [cédérom]. 2nd edn FNCLCC, John Libbey EUROTEXT, Paris

Langmark F (1997) Cancer in Norway: the cancer registry of Norway. Norwegian Cancer Registry: Oslo, pp 98–99

Lee SJ, Earle CC and Weeks JC (2000) Outcomes research in oncology: history, conceptual framework, and trends in the literature. *J Natl Cancer Inst* **92**: 195–204

LeLorier J, Gregoire G, Benhaddad A, Lapierre J and Derderian F (1997) Discrepancies between meta-analyses and subsequent large randomized, controlled trials [see comments]. *N Engl J Med* **337**: 536–542

Marshall E (2000) Biomedical ethics. Penn report, agency heads home in on clinical research [news]. *Science* **288**: 1558–1559

Organization of European Cancer Institutes (2000) 20th anniversary report 1979–1999. Brno, Czech Republic

Quinn MJ, Martinez-Garcia C and Berrino F (1998) Variations in survival from breast cancer in Europe by age and country, 1978–1989. *Eur J Cancer* **34**: 2204–2211

Therasse P, Arbuck SG, Eisenhauer EA, Wanders J, Kaplan RS, Rubinstein L, Verweij J, Van Glabbeke M, van Oosterom AT, Christian MC and Gwyther SG (2000) New guidelines to evaluate the response to treatment in solid tumors. European Organization for Research and Treatment of Cancer, National Cancer Institute of the United States, National Cancer Institute of Canada [see comments]. *J Natl Cancer Inst* **92**: 205–216

United Kingdom Co-ordinating Committee on Cancer Research (2000) UKCCCR guidelines for the use of cell lines in cancer research. *Br J Cancer* **82**: 1495–1509

*© 2001 FNCLCC*
